# Adipose-derived stem cell-based optimization strategies for musculoskeletal regeneration: recent advances and perspectives

**DOI:** 10.1186/s13287-024-03703-6

**Published:** 2024-03-27

**Authors:** Chenrui Yuan, Wei Song, Xiping Jiang, Yifei Wang, Chenkai Li, Weilin Yu, Yaohua He

**Affiliations:** 1 Department of Orthopedics, Shanghai Sixth People’s Hospital Affiliated to Shanghai Jiao Tong University School of Medicine, Shanghai, 200233 China; 2 Department of Orthopedics, Jinshan Branch of Shanghai Sixth People’s Hospital Affiliated to Shanghai University of Medicine & Health Sciences, Shanghai, 201500 China

**Keywords:** Adipose-derived stem cells, Exosomes, Bone regeneration, Cartilage regeneration, Tendon regeneration, Musculoskeletal disorders, Optimization strategies

## Abstract

Musculoskeletal disorders are the leading causes of physical disabilities worldwide. The poor self-repair capacity of musculoskeletal tissues and the absence of effective therapies have driven the development of novel bioengineering-based therapeutic approaches. Adipose-derived stem cell (ADSC)-based therapies are being explored as new regenerative strategies for the repair and regeneration of bone, cartilage, and tendon owing to the accessibility, multipotency, and active paracrine activity of ADSCs. In this review, recent advances in ADSCs and their optimization strategies, including ADSC-derived exosomes (ADSC-Exos), biomaterials, and genetic modifications, are summarized. Furthermore, the preclinical and clinical applications of ADSCs and ADSC-Exos, either alone or in combination with growth factors or biomaterials or in genetically modified forms, for bone, cartilage, and tendon regeneration are reviewed. ADSC-based optimization strategies hold promise for the management of multiple types of musculoskeletal injuries. The timely summary and highlights provided here could offer guidance for further investigations to accelerate the development and clinical application of ADSC-based therapies in musculoskeletal regeneration.

**Graphical abstract:**

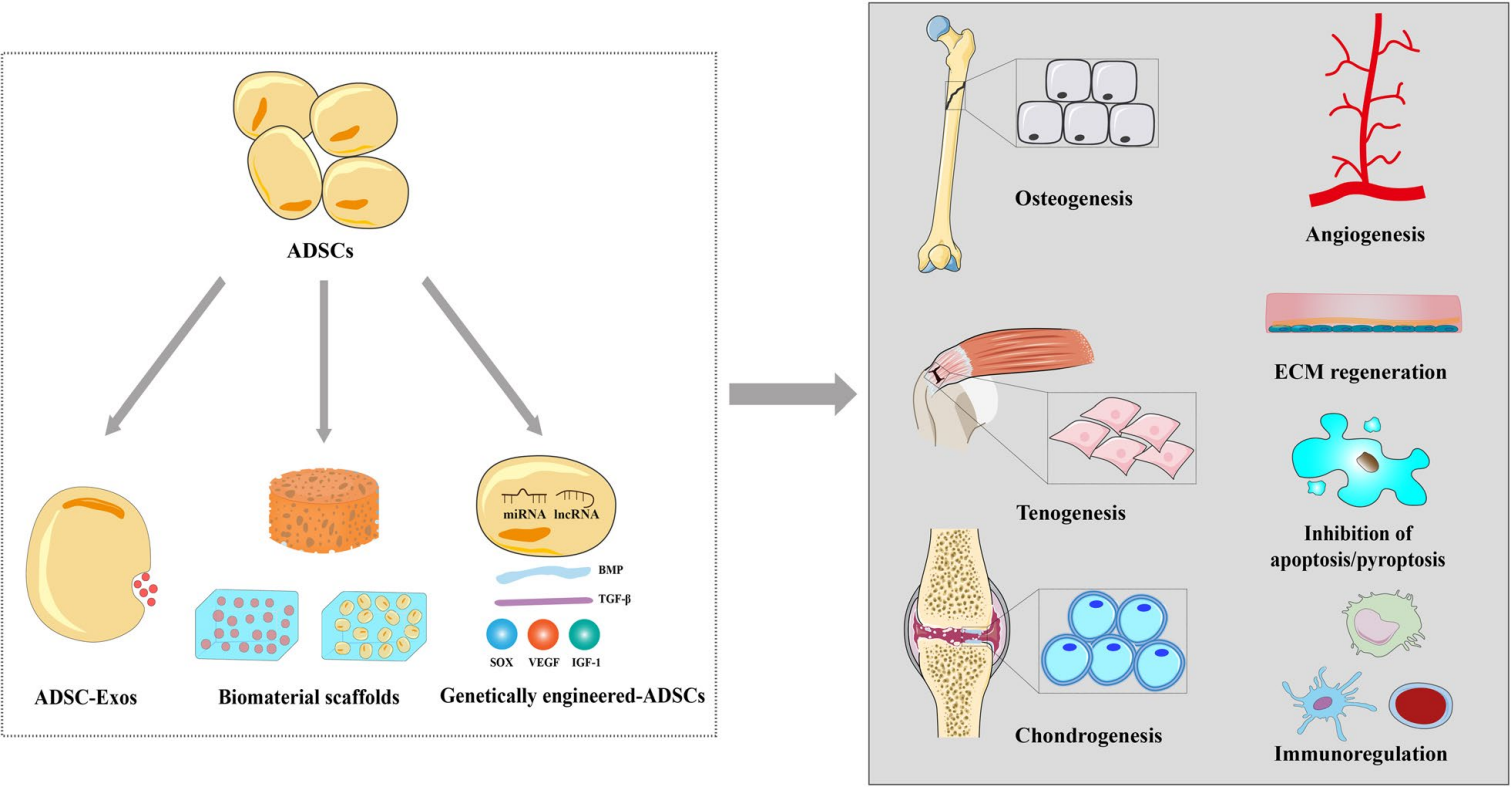

## Background

Musculoskeletal disorders that affect bone, cartilage, tendons, ligaments, and skeletal muscles are characterized by tissue degeneration, debilitating pain, functional impairment, and disability. These disorders impact 1.7 billion people worldwide, with the burden doubling over the last few decades owing to factors such as aging, obesity, traumatic injuries, and genetic mutations [1]. Musculoskeletal tissues such as tendons and ligaments have a limited self-repair capacity, whereas articular cartilage exhibits poor intrinsic regenerative potential. Bones and skeletal muscles possess the ability to regenerate after minor injuries. However, larger or chronic injuries, including critical-sized bone defects and pathological fractures, can exceed the self-repair capacity of these tissues [2]. These characteristics contribute to the development and progression of musculoskeletal diseases.

Tissue engineering and regenerative medicine are the focus of research aimed at repairing or replacing defective tissues and restoring their biological functions, and such work can potentially provide solutions to the growing prevalence of musculoskeletal disorders. These approaches typically integrate stem cells, engineering materials, and bioactive molecules. Researchers have engineered and delivered cells with regenerative capacity to damaged tissues, which represents the basis for diverse bioengineering approaches for musculoskeletal regeneration [3].

Adipose tissue, derived from the embryonic mesoderm, is a valuable cell source for tissue engineering. Adipose-derived stem cells (ADSCs) were first characterized in 2001 [4]. As an important subpopulation of mesenchymal stem cells (MSCs), ADSCs have the potential for multidirectional differentiation, regeneration, and multiple cytokine secretions. Compared with MSCs derived from bone marrow (BM) or other sources, ADSCs have the advantages of accessibility, abundance, and reduced donor site morbidity [5]. Adipose tissue yields approximately 500 times more stem cells compared to an equivalent amount of BM, with approximately 5000 ADSCs obtained per 1 g of adipose tissue [6]. Hence, large amounts of ADSCs can be obtained from adipose tissue via minimally invasive procedures. Moreover, ADSCs exhibit a higher proliferation rate and better colony-forming potency than BM-MSCs under similar culture conditions [7]. Single-cell RNA sequencing analysis indicates that ADSCs exhibit lower transcriptomic heterogeneity, lower human leukocyte antigen (HLA) class I expression, and higher immunosuppressive capacity than BM-MSCs [8]. In addition, ADSCs have low immunogenicity and immune-privileged potential owing to the absence or low expression of HLA and co-stimulatory molecules [9, 10]. Moreover, unlike embryonic stem cells, there are no ethical issues surrounding the acquisition of ADSCs. All these properties indicate that ADSCs represent an interesting and feasible alternative to BM-MSCs or other types of MSCs. Therefore, they are considered prominent candidates for cellular bioengineering and hold promising prospects in tissue repair and regeneration.

In recent decades, ADSCs have been increasingly explored for their regenerative potential in various conditions, including wound healing, neurodegenerative diseases, cardiovascular diseases, and liver cirrhosis [11–16]. Furthermore, ADSC-based and related tissue engineering strategies are being investigated for bone, cartilage, and tendon repair and regeneration [3, 17–21]. Hence, in this study, we review ADSCs and novel optimization strategies for improving their regenerative potential, including ADSC-derived exosomes (ADSC-Exos), biomaterials, and genetically engineered modifications. Next, we discuss the preclinical and clinical applications of ADSCs and ADSC-Exos, either alone or in combination with growth factors or biomaterials or in a genetically modified form, in bone, cartilage, and tendon regeneration in various musculoskeletal disorders. With this review, we hope to provide further insights into the vital roles and applications of ADSCs in musculoskeletal tissue engineering and regenerative medicine. To achieve this, we searched PubMed, Web of Science, and EMBASE using “adipose-derived stem cells” or “ADSCs” and the three related topics mentioned above as keywords to retrieve the relevant orthopedic studies published over the past 5 years. We focused on studies involving cell and animal experiments or clinical trials, particularly original articles related to the topic.

## ADSC characteristics

Apart from being an energy reservoir for the body, adipose tissue acts as an endocrine organ that regulates the body’s metabolism and immunity [22]. There are two types of adipose tissues, i.e., white and brown, and they differ in terms of their distribution, function, and metabolic activity. White adipose tissue is predominantly involved in lipid storage and serves as a source of ADSCs. Brown adipose tissue, a key regulator of thermogenesis, is predominantly present in fetuses and newborns and declines with age [23]. Stromal vascular fraction (SVF) is conventionally isolated from the enzymatic digestion of white adipose tissue and represents a heterogeneous cell group consisting of preadipocytes, vascular smooth muscle cells, endothelial cells, monocytes/macrophages, lymphocytes, fibroblasts, pericytes, and ADSCs. ADSCs account for 15–30% of the SVF [24]. Owing to their plastic adherence capacity, ADSCs can be easily harvested from the SVF in a culture system.

However, ADSCs are not completely homogeneous, and there is no single biomarker to explicitly characterize them. In 2013, the International Federation for Adipose Therapeutics and Science (IFATS) and the International Society for Cellular Therapy (ISCT) described the phenotypic and functional criteria for ADSCs [24]. Phenotypic identification includes primary positive markers, such as cluster of differentiation (CD)13, CD29, CD44, CD73, CD90, and CD105, and primary negative markers, such as CD31, CD45, and CD235a. Additional secondary positive markers include CD10, CD26, CD36, CD49d, and CD49e, whereas secondary low or negative markers include CD3, CD11b, CD49f, CD106, and podocalyxin-like protein. CD34 is the primary unstable marker present at various levels in ADSCs. It is generally expressed during the early culture phase (within 8–12 population doublings after SVF culture); however, its expression decreases with continued cell division [24, 25]. Moreover, the IFATS and ISCT provided recommendations for the functional assessment of ADSCs. The fibroblastoid colony-forming unit assay is generally used to calculate the population-doubling capacity of ADSCs. Chondroblastic, osteoblastic, and adipocytic differentiation assays have been employed to assess the differentiation potential of ADSCs. Furthermore, quantitative evaluations of lineage-specific gene or protein biomarkers using biochemical methods (western blotting, enzyme-linked immunosorbent assay) or reverse transcription polymerase chain reaction have been proposed to characterize the differentiation potential of ADSCs. The following biomarkers have been proposed: aggrecan, collagen type II, and Sox9 for chondrogenesis; alkaline phosphatase, bone sialoprotein, osteocalcin, osterix, and Runx2 for osteogenesis; and collagen III, tenomodulin, and tenascin C for tenogenesis [24, 26–28]. Recent advances in single-cell technology provide new methods of identifying the multidirectional differentiation potential of ADSCs [29, 30].

**Fig. 1:**
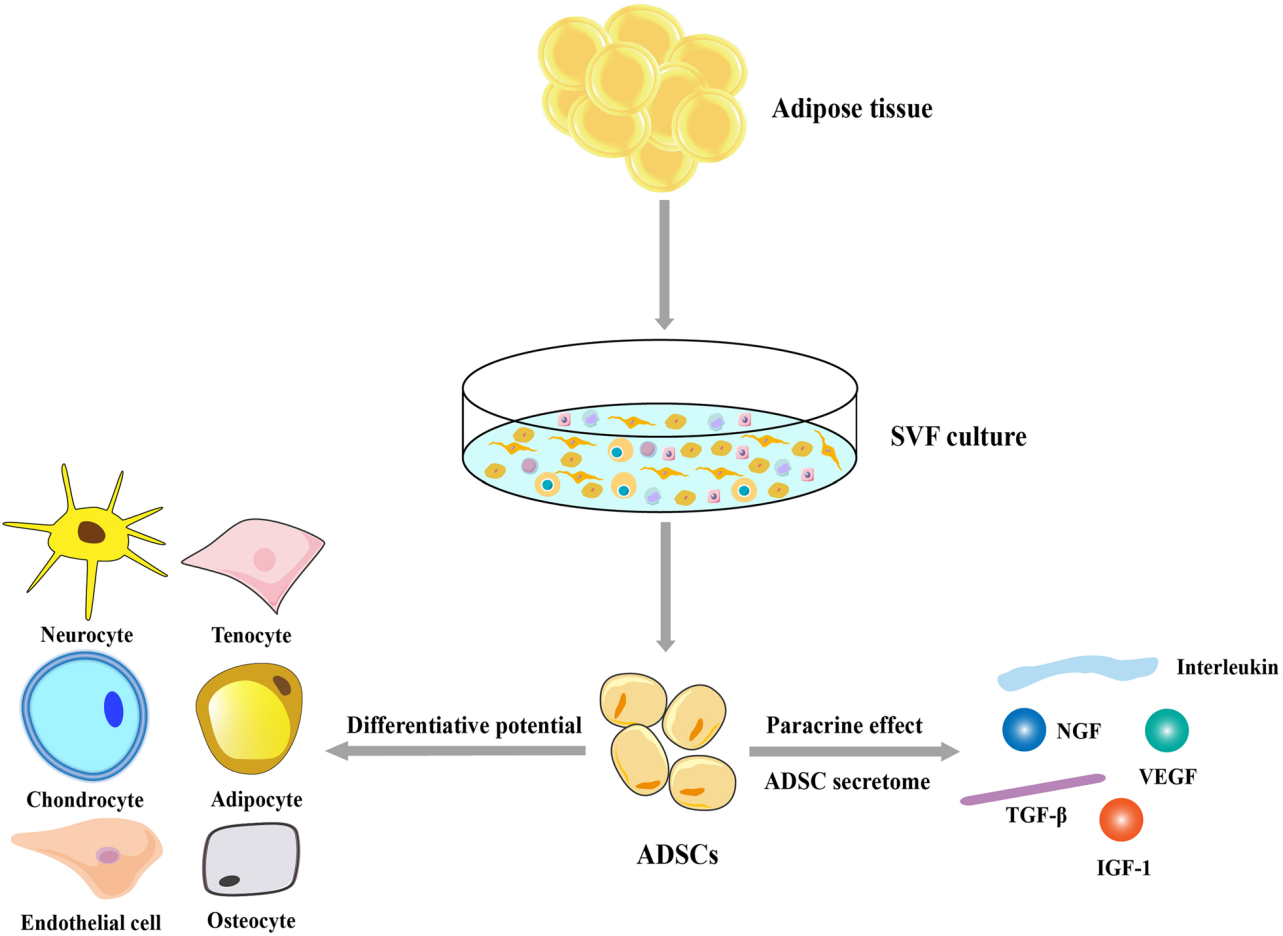
Differentiation potential and paracrine activity of ADSCs. ADSCs can be harvested as part of the SVF, which is traditionally isolated from the enzymatic digestion of the adipose tissues. ADSCs exhibit regenerative abilities and can differentiate into specific cell types in the presence of lineage-specific induction factors and secrete multiple bioactive molecules, which constitute the “secretome” profile. ADSCs: adipose-derived stem cells; IGF-1: insulin-like growth factor 1; NGF: nerve growth factor; SVF: stromal vascular fraction; TGF-β: transforming growth factor-β; VEGF: vascular endothelial growth factor

ADSCs can differentiate in vitro and in vivo into osteogenic, chondrogenic, myogenic, epithelial, endothelial, and neuron-like cells in the presence of lineage-specific induction factors [31]. In addition, mechanical or electromagnetic stimulation, pharmaceuticals, and genetic reprogramming have been widely explored to promote the differentiation of ADSCs into specific cell lineages [32, 33]. However, the therapeutic potential of ADSCs is not limited to the specific differentiation and replacement of defective cells. The “secretome” theory is based on the ability of ADSCs to secrete multiple cytokines, growth factors, chemokines, and antioxidant factors [e.g., vascular endothelial growth factor (VEGF), insulin-like growth factor-1 (IGF-1), nerve growth factor (NGF), hepatocyte growth factor, and multiple interleukins (ILs)] alone or as cell-free extracellular vesicles (EVs) [34–36] (Fig. 1), while the “building block” or “host replacement” theories indicate that ADSCs can differentiate and replace damaged cells. Studies on the secretome profile have also indicated that the paracrine activity of the secretome may regulate various biological effects, including angiogenesis, apoptosis suppression, and immunoregulation [16, 17, 36–38]. Thus, the ADSC secretome may be an important therapeutic mediator necessary in tissue repair and regeneration.

## Optimization of ADSC-based therapeutic strategies

### ADSC secretome

As mentioned above, ADSCs exert regenerative effects not only by direct cell replacement but also via the secretome, including EVs and other bioactive molecules. Considering the numerous limitations associated with the direct transplantation of ADSCs, such as the need for strict monitoring during production and storage and the potential risks of tumorigenicity and immune rejection, the “cell-based but cell-free” therapy approach utilizing the ADSC secretome has recently gained considerable attention as an alternative strategy. During cell culture, ADSCs typically release various bioactive molecules into the medium, which is known as the conditioned medium (CM). ADSC-CM consists of a complex cocktail of proteins, nucleic acids, and lipids released as active factors and/or conveyed to EVs, collectively constituting the entire ADSC secretome and contributing to the paracrine effects of ADSCs [39]. Studies have demonstrated the beneficial effects of ADSC-CM on wound healing [40], skin photoaging [41], and cardioprotection [16]. EVs are a heterogeneous group of cell-derived lipid bilayer membranes that can be categorized into various subclasses, including exosomes, microvesicles, and apoptotic bodies [42]. In recent years, ADSC-Exos extracted from ADSC-CM has emerged as the focus of ongoing investigations in regenerative medicine.

### ADSC-Exos

Exosomes are the most abundant and well-characterized type of EVs and have diameters ranging between 40 and 160 nm [43]. They originate within endosomal compartments and encapsulate specific proteins, nucleic acids (mRNAs, miRNAs, and long noncoding RNAs [lncRNAs]), lipids, amino acids, and metabolites within their lipid bilayer membranes. Upon release into the extracellular space, they are transported to target cells and participate in intercellular communication [43]. ADSC-Exos, which are naturally secreted by ADSCs, can promote angiogenesis, regulate immune and inflammatory responses, affect collagen remodeling, inhibit cell apoptosis, and promote tissue repair and regeneration. These functions stem from the diverse proteins and genetic materials encapsulated within them [44–46]. Compared with ADSCs, ADSC-Exos are smaller and less complex than their parent cells, and they are also more stable and convenient to store. Additionally, their dosage can be easily controlled [47]. Functionally, ADSC-Exos have low immunogenicity and can reduce immune recognition to maintain cell membrane integrity [48].

**Fig. 2:**
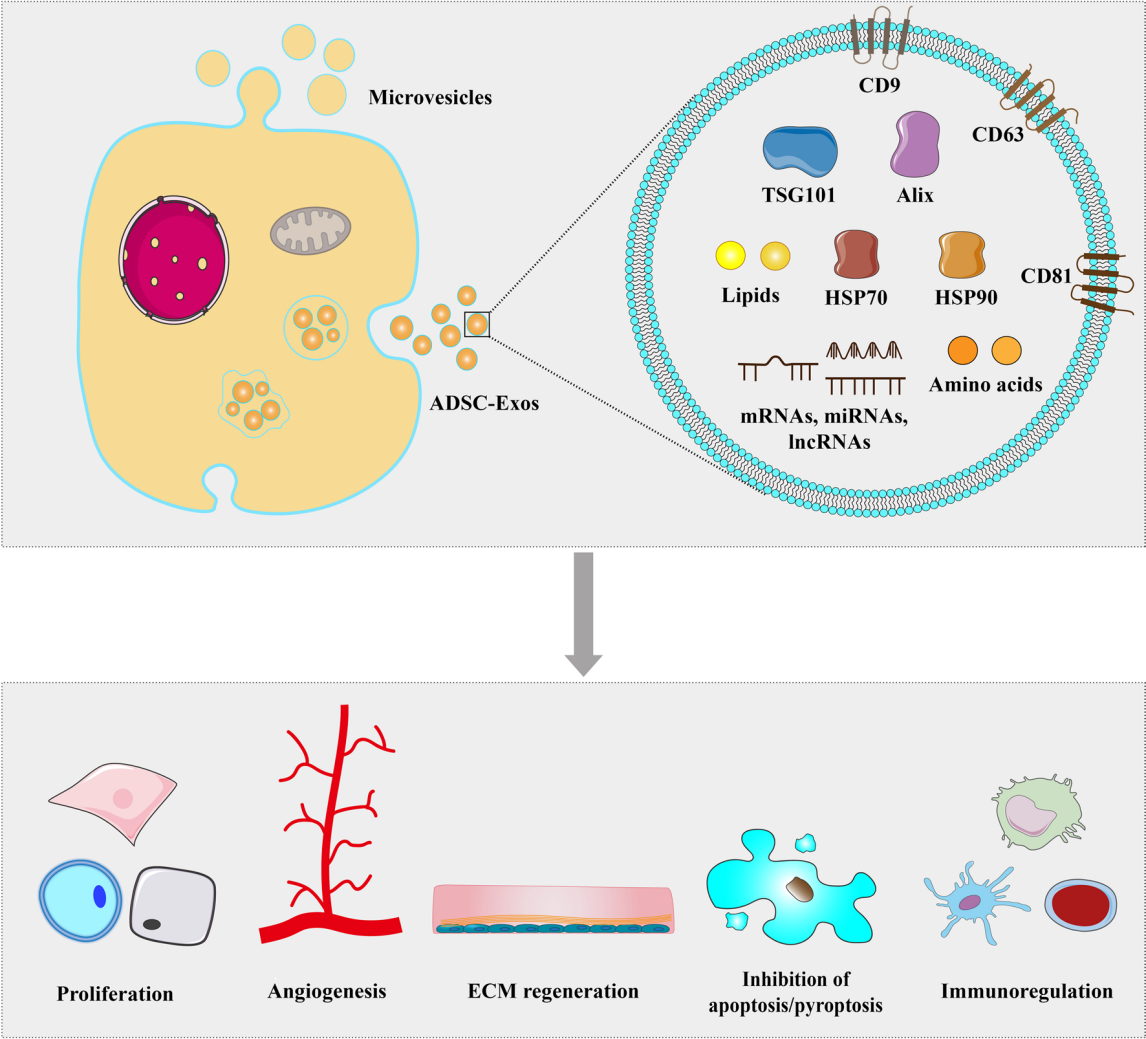
Characteristics and therapeutic potential of ADSC-Exos. ADSC-Exos contain tetraspanins (CD9, CD63, and CD81), vesicle-forming proteins (Alix and TSG101), heat shock proteins (HSP70 and HSP90), nucleic acids (mRNAs, miRNAs, and lncRNAs), lipids, and amino acids. ADSC-Exos exert their therapeutic potential by enhancing cell proliferation, angiogenesis, and ECM synthesis, participating in immunoregulation, and inhibiting apoptosis or pyroptosis. ADSC-Exos: adipose-derived stem cell-derived exosomes; ECM: extracellular matrix; HSP: heat shock protein; TSG: tumor suppressor gene

Several methods are available for harvesting exosomes. Among them, ultracentrifugation is the most commonly used and considered the gold standard for isolation. Ultrafiltration can be used both as a standalone isolation approach and as a complement to ultracentrifugation. In addition to these traditional approaches, innovative technologies and new platforms, such as a magnetic bead-based adsorption strategy, have emerged for exosome isolation [49]. During the biosynthesis of ADSC-Exos, a range of proteins are enriched, among which tetraspanins (CD9, CD63, and CD81), vesicle-forming proteins (Alix and tumor suppressor gene 101 [TSG101]), and heat shock proteins (HSP70 and HSP90) are generally recognized as characteristic biomarkers [50] (Fig. 2). Furthermore, electron microscopy and nanoparticle tracking analysis can be used to identify ADSC-Exos. Multiomics has recently been used to identify specific marker proteins in exosomes [51, 52]. This advancement may provide a novel method to elucidate the functions of ADSC-Exos in the future. Thus far, the therapeutic effects of ADSC-Exos have been demonstrated in the field of regenerative medicine [53, 54]. ADSC-Exos combined with optimization strategies, including exosome incorporation into biomaterials [55] or genetic modification [56], represents a feasible strategy for further application of ADSC-Exos.

### Biomaterial scaffolds

An ideal biomaterial scaffold can mimic the natural extracellular environment and provide mechanical support to facilitate the delivery, proliferation, migration, and differentiation of ADSCs [3]. Moreover, incorporating ADSC-Exos into suitable biomaterials can help control their release, distribution, and retention in vivo, thereby enhancing their therapeutic function [57]. Thus, the combination of ADSCs or exosomes with novel scaffold materials is a promising alternative administration method.

**Fig. 3:**
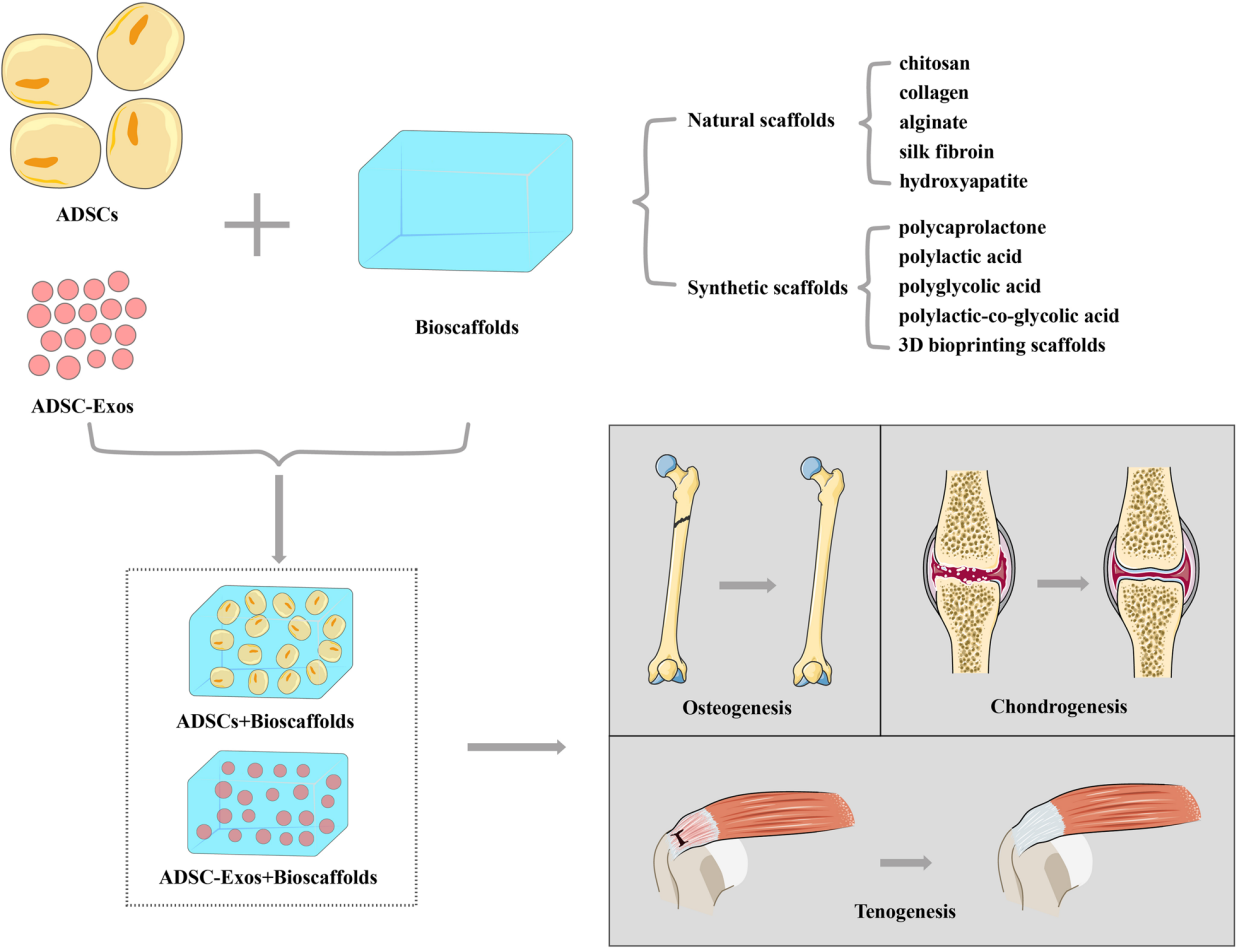
ADSCs or ADSC-Exos combined with biomaterial scaffolds for musculoskeletal regeneration. ADSCs: adipose-derived stem cells; ADSC-Exos: adipose-derived stem cell-derived exosomes

In musculoskeletal tissue engineering, scaffold materials include organic and inorganic sources, such as natural polymers (e.g., fibrin, hyaluronic acid, chitosan, collagen, alginate, and silk fibroin), inorganic materials (e.g., hydroxyapatite, tricalcium phosphate, glass ceramics, and titanium), and synthetic biodegradable polymers [e.g., polycaprolactone, polylactic acid (PLA), polyglycolic acid, and polylactic-co-glycolic acid (PLGA)] [58, 59]. Novel synthetic scaffolds can be fabricated from different biomaterials, such as natural and synthetic polymers or inorganic ceramics and polymers, thereby eliminating the disadvantages of conventional scaffolds while enhancing their properties, including mechanical strength, porosity, wettability, angiogenic potential, and cell-material interactions required for tissue regeneration [60, 61]. Decellularized extracellular matrix (ECM)-based biomaterials, such as injectable hydrogels and electrospun scaffolds, have also been developed to provide structural support, act as local reservoirs of growth factors, and provide biochemical cues to guide ADSC differentiation and regeneration [62, 63]. Three-dimensional (3D) bioprinting is an advanced strategy for replicating the functional organization of human tissues and simulating 3D micro-tissue environments. Through 3D bioprinting, various biomaterials can be built layer-by-layer to fabricate bioengineered constructs with predefined dimensions and spatial distributions. Combination of 3D-printed scaffolds with ADSCs or ADSC-Exos can be used to generate zonal distributions of cells/exosomes, matrix proteins, and bioactive cues; construct an optimal biomimetic environment; and promote the simultaneous regeneration of different musculoskeletal tissues [55, 64, 65] (Fig. 3).

### Genetically engineered ADSCs

Genetic modification of ADSCs can enable the sustained localized expression of specific genes, thereby avoiding the off-target effects associated with systemic delivery or burst release and subsequently enhancing their therapeutic potential, including proliferation, lineage-specific differentiation, or immunoregulation. Most studies using genetically modified ADSCs have involved osteoinductive bone morphogenetic proteins (BMPs), which are members of the transforming growth factor-β (TGF-β) family and represent the most effective factors in regulating bone induction, maintenance, and repair [66]. TGF-β [67], IGF-1 [68], and the transcription factor SOX [69] have been genetically engineered with ADSCs for the stimulation of chondrogenic differentiation and synthesis of cartilage-specific matrix components and VEGF for triggering angiogenesis [70]. Furthermore, genetically modified ADSCs can be embedded within biomaterial scaffolds to increase cell survival and retention at implantation sites [71].

In addition to genetic transformation to express specific protein-coding genes, epigenetic regulation plays an important role in the induction of ADSC differentiation. MicroRNAs (miRNAs) are a class of small noncoding RNAs (ncRNAs) that act as post-transcriptional regulators of gene expression, and they are associated with the regulation of several cellular processes, including proliferation, migration, and differentiation [72]. Thus, miRNA mimics, anti-/antago-miRNAs, or vectors that overexpress or suppress miRNA levels have been used to modulate ADSCs for osteochondral regeneration [73–75]. Moreover, another class of ncRNAs called lncRNAs has been explored as a mediator for inducing osteogenesis in ADSCs [76, 77].

In general, the abovementioned genetically engineered ADSC-based therapies represent promising approaches for enhancing the therapeutic potential of ADSCs. However, the consistent activation of specific genes may be a risk factor for stem cell-derived tumors. Hence, in clinical practice, the application of genetic modifications must be approached cautiously and needs further validation.

## ADSCs and ADSC-based therapies for musculoskeletal regeneration

Based on the potential of ADSCs for multidirectional differentiation, regeneration, and paracrine activity, they can be applied in novel bioengineering approaches to address the requirement for musculoskeletal regeneration in various musculoskeletal disorders, including bone defects, non-union fractures, osteoarthritis, tendon injuries, and intervertebral disk degeneration (IDD).

### Bone regeneration

Bone tissue has inherent repair and regeneration capacities. However, defective bone tissue cannot recover its integrity when extensive or chronic injuries occur, such as critical-sized bone defects and bone non-unions, which may be caused by heavy trauma, osteomyelitis, bone tumor resection, congenital malformations, and prosthesis revision, or when the regenerative process is impaired owing to factors such as age, comorbidities, and genetic factors [78]. The reconstruction of bone defects or bone non-unions usually requires invasive bone transfer or foreign body implants, which may result in secondary donor site morbidity and an increased risk of infection and extrusion. Bone tissue engineering can promote bone healing without encountering these disadvantages. Notably, ADSC-based therapies represent a promising alternative for bone regeneration (Table 1).

**Table 1:** Recent preclinical studies on ADSC-based optimization strategies for bone regeneration

ADSC-based optimization strategies		Models	Methods	Results	References
Bioscaffolds	Heterogeneous deproteinized bone (HDB)	Rat radial defect model	ADSCs seeded onto the HDB were implanted into the defective area	ADSC-HDB composites exhibited a strong osteogenic ability	Liu et al. [89]
	Modified hierarchical mesoporous bioactive glass (MBG) scaffold	Rat femoral defect model	Osteogenically induced ADSCs were seeded on the MBG scaffolds prevascularized by endothelial-induced ADSCs; the composite scaffolds were implanted into the defective area	Time-phased sequential application of ADSCs on the MBG scaffolds promoted better angiogenesis and mineral deposition	Du et al. [90]
Genetic modification + Bioscaffolds	Transduction of BMP-2; Biofabricated cryogel scaffolds	Mouse atrophic non-union model	ADSCs were seeded onto biofabricated cryogel scaffolds after BMP-2 transduction and implanted into the non-union site	ADSC-seeded cryogels promoted osseous healing	Hixon et al. [94]
	miR-150-5p inhibition; Hydroxyapatite/tricalcium phosphate (HA/TCP) ceramic powder	BALB/c nu/nu mice	miR-150-5p-modified ADSCs were loaded in HA/TCP ceramic powders and implanted into the dorsal surface of BALB/c nu/nu mice	Combination of ADSCs, miR-150-5p inhibition, and HA/TCP promoted bone damage repair and bone regeneration	Wang et al. [95]
Engineered ADSC spheroids		Mouse calvarial defect model	ADSCs were assembled with PDGF and biomineral-coated fibers to form spheroids and implanted into the defective area	ADSC spheroids incorporating PDGF and biominerals exhibited greater endothelial lineage mRNA expression and vascularized bone regeneration	Lee et al. [91]
		Mouse calvarial defect model	ADSCs were assembled with adenosine and polydopamine-coated fibers to form spheroids and implanted into the defective area	ADSC spheroids impregnated with engineered fibers enabled adenosine delivery and promoted bone regeneration with enhanced osteogenic differentiation	Ahmad et al. [92]
ADSC-Exos + Bioscaffolds	Gelatin nanoparticles (GNPs)	Rat skull defect model	ADSC-Exos loaded within GNPs were implanted into the defective area	GNP-ADSC-Exos effectively regulated bone immune metabolism and promoted bone healing partly via the immune regulation of miR-451a	Li et al. [17]
	Metal–organic framework (MOF) scaffolds	Rat calvarial defect model	ADSC-Exos were coated on the PLGA/Mg-GA MOF scaffold and implanted into the defective area	PLGA/Exo-Mg-GA MOF scaffolds promoted osteogenesis and satisfactory osseointegration	Kang et al. [57]
	PLGA/pDA scaffolds	Mouse calvarial defect model	ADSC-Exos were immobilized on PLGA/pDA scaffolds and then implanted into the defective area	Composite of ADSC-Exos and PLGA/pDA scaffolds enhanced bone regeneration, partially via their osteoinductive effects and by promoting stem cell migration and homing	Li et al. [96]
	Gelatin sponge/polydopamine (GS-PDA) scaffolds	Rat femoral defect model	ADSC-Exos-modified GS-PDA scaffolds (GS-PDA-Exos) were implanted into the defective area	GS-PDA-Exos promoted osteogenesis and bone repair	Li et al. [99]
ADSC-Exos + Genetic modification + Bioscaffolds	Exosomes derived from genetically modified ADSCs; Hydrogel comprising thiol‐modified hyaluronan, hydroxyapatite, and thiol‐modified heparin	Rat calvarial defect model	Exosomes derived from miR-375-overexpressing ADSCs were embedded in hydrogels and implanted into the defective area	ADSC-Exos enriched with miR-375 could enhance bone regeneration	Chen et al. [56]

ADSCs can differentiate into the osteogenic lineage when exposed to differentiation media containing BMPs [79], platelet-rich plasma [80], vitamin D3 [81], and human platelet lysates [82]. BMPs are the most effective osteoinductive factors. They interact with pathways such as the Smad, mitogen-activated protein kinase, Wnt, and Sonic Hedgehog pathways to regulate osteogenesis [83]. The abovementioned agents may serve as potential candidates to accelerate the osteogenic differentiation of ADSCs. The bone regenerative effects of ADSCs were explored in a murine model with large bony defects resulting from osteomyelitis, and ADSCs were locally administered to the damaged area after sufficient debridement of the infected bones. ADSCs promoted bone healing by increasing osteoblastogenesis and decreasing osteoclast and B cell numbers, thereby highlighting the bone regeneration potential of ADSCs in post-infectious inflammatory states [84].

One strategy to enhance the osteogenic potential of ADSCs involves the use of biomaterials and/or matrix proteins to recreate a precursor cell niche that promotes cell adhesion, proliferation, and differentiation [85, 86]. ADSCs have been successfully combined with biomaterials such as bioactive glass [87], tricalcium phosphate, fibronectin [88], and heterogeneous deproteinized bone [89] to improve bone regeneration in animal models. Du et al. designed a composite that utilized the multilineage differentiation capacity of ADSCs into osteogenic and vascular cells and seeded the endothelial-induced ADSCs into a mesoporous bioactive glass (MBG) scaffold [90]. This prevascularized MBG scaffold was combined with osteogenically induced ADSCs to repair critical-size bone defects in a rat model. The time-phased sequential application of ADSCs promoted better angiogenesis and mineral deposition compared to nonvascularized MBG-carrying osteogenically induced ADSCs, attributed to the rapid angiogenesis and improved survival of seeded ADSCs [90]. Recently, engineered stem cell spheroids have demonstrated great potential for bone regeneration. For example, Lee et al. assembled ADSCs with platelet-derived growth factor (PDGF) and biomineral-coated fibers to build ASDC spheroids that exhibited high mRNA expression associated with the endothelial lineage and vascularized bone regeneration [91]. Similarly, Ahmad et al. assembled ADSCs with adenosine and polydopamine-coated fibers to engineer ADSC spheroids, which enabled adenosine delivery and promoted bone regeneration by enhancing osteogenic differentiation [92]. In summary, engineered ADSC spheroids are promising alternatives for bone regeneration.

Genetic modifications of ADSCs to promote angiogenesis and osteogenic differentiation have been verified in a mouse model of femoral fracture [93]. Furthermore, genetically engineered ADSCs combined with bioscaffolds have been explored as an optimization strategy. Hixon et al. seeded murine ADSCs onto biofabricated cryogel scaffolds after the lentiviral transduction of BMP-2 and surgically implanted the seeded scaffolds around the non-union site in a 3.6Col1A1-tk (Col1-tk) mouse model. The ADSC-seeded cryogel scaffolds promoted bone formation while enhancing healing [94]. miRNAs play synergistic or antagonistic roles in regulating cell differentiation. Recently, Wang et al. demonstrated that miR-150-5p inhibition can increase ADSC osteogenesis by regulating Notch3 and that the combination of ADSCs, miR-150-5p inhibitors, and hydroxyapatite/tricalcium phosphate ceramic powders can enhance bone regeneration and bone damage repair [95].

The therapeutic effects of the ADSC secretome on bone repair and regeneration have been validated in several preclinical studies. In vitro, ADSC-Exos effectively antagonizes osteocyte apoptosis by promoting anti-apoptotic Bcl-2 expression and suppressing pro-apoptotic Bax expression [46]. The combination of ADSC-Exos with novel biomaterials can enhance bone regeneration. ADSC-Exos modified with efficient biocompatible carriers, such as polydopamine (pDA)-coated PLGA [96], alginate-cobalt ferrite [97], mineral-doped porous scaffolds constructed with PLA, calcium silicates, and dicalcium phosphate dihydrate [98], and gelatin sponge/polydopamine scaffolds [99], can improve osteogenesis and repair bone defects. Kang et al. synthesized a metal–organic framework (MOF) using human ADSC-Exos, PLGA, Mg^2+^, and gallic acid (PLGA/Exo-Mg-GA MOF) to create unique nanostructural interfaces that could enhance the osteogenic, angiogenic, and anti-inflammatory capabilities of ADSC-Exos [57]. Furthermore, the bone formation enhancement effect of ADSC-Exos loaded with effective osteogenic agents has been assessed. Lu et al. demonstrated that exosomes derived from tumor necrosis factor (TNF)-α-pre-conditioned ADSCs could enhance human osteoblastic proliferation and differentiation in vitro by activating Wnt signaling, achieved by increasing Wnt-3a levels within the exosomes [100]. Exosomes derived from miR-375-overexpressing human ADSCs embedded in hydrogels could deliver miRNAs to human BMSCs. This composite inhibited the expression of IGF-binding protein 3 and subsequently promoted bone regeneration [56]. Overall, these studies suggest that ADSC-Exos have considerable potential for use in bone tissue engineering.

Encouraging results from preclinical studies on ADSCs have prompted multiple clinical trials. The majority of these trials focused on treating craniomaxillofacial bone injuries or defects and achieved generally meaningful clinical effects. Defects were reconstructed using either bioactive glass or β-tricalcium phosphate (β-TCP) scaffolds seeded with ADSCs and, in some cases, combined with recombinant human BMP-2 [101]. The transplantation of autologous human ADSCs did not result in major adverse events, the focal bone formation was good, and a few patients achieved excellent clinical results. However, problems surrounding graft loosening, infection, and tumor recurrence were observed [102]. A 6-year follow-up study of cranioplasty based on ADSCs, β-TCP, and a supporting mesh illustrated unsatisfactory results, such as marked graft resorption, late infection (7.3 years postoperatively), or meningioma recurrence (2.2 years postoperatively). These observations indicate that ADSC-based treatment is not superior to conventional cranial repair methods [103]. Despite the potential of ADSCs in bone repair and regeneration, difficulties and barriers remain in clinical trials. Hence, the clinical application of ADSCs for bone defects requires further investigation.

### Cartilage regeneration

Cartilage damage caused by acute trauma or chronic degeneration predominantly presents as joint lesions. Owing to the poor intrinsic regenerative potential of human cartilage tissue, these damages can progress to osteoarthritis or necessitate arthroplasty [104]. Current management of cartilage defects, particularly osteoarthritis, only provides symptom relief rather than a cure. Recently, cartilage tissue engineering, which integrates engineering and biological approaches to promote cartilage regeneration and damaged tissue replacement, has become an important research area. Stem cell therapies, including ADSCs and ADSC-based optimization strategies, provide promising new alternatives for treating cartilage defects (Table 2).

**Table 2:** Recent preclinical studies on ADSC-based optimization strategies for cartilage regeneration

ADSC-based optimization strategies		Models	Methods	Results	References
ADSCs		Rat osteoarthritis model	ADSCs were injected into the articular cavity	ADSCs inhibited the expression of NLRP3, Caspase1, GSDMD, and TNFR1; improved the pathological changes in the joints; and delayed osteoarthritis development	Xu et al. [107]
ADSC-EVs		Rat osteoarthritis model	ADSC-EVs were injected into the articular cavity	ADSC-EVs protected the cartilage from degeneration, inhibited the infiltration of M1 macrophages into the synovium, and attenuated osteoarthritis progression	Woo et al. [21]
ADSC-Exos	Hypoxic pre-condition of ADSCs	Rat model of post-traumatic osteoarthritis	Intra-articular injection of hypoxia-cultured ADSC-secreted exosomes (hypoxia-ADSC-Exos)	Hypoxia-ADSC-Exos exhibited a chondroprotective effect and suppressed osteoarthritis progression	Chang et al. [114]
	Tropoelastin pretreatment of ADSCs	Rat model of osteoarthritis induced by the anterior cruciate ligament transection (ACLT)	Intra-articular injection of exosomes from tropoelastin-pretreated ADSCs (TE-ADSCs-Exos)	TE-ADSCs-Exos enhanced matrix synthesis and promoted cartilage repair	Meng et al. [115]
Bioscaffolds	Biomimetic injectable hydrogel using amnion membrane (AM)	Collagenase-induced osteoarthritis rat model	Intra-articular injections of AM hydrogels with ADSCs	AM hydrogels with or without ADSCs alleviated inflammation and cartilage degeneration	Bhattacharjee et al. [18]
	Glycol chitosan/dibenzaldehyde- functionalized- polyethylene glycol (GCS/DF-PEG) hydrogel	Rat model of articular cartilage defect	ADSCs with GCS/DF-PEG hydrogel were injected into the defective area	ADSCs and GCS/DF-PEG hydrogel complexes exhibited obvious cartilage regeneration ability	Yang et al. [119]
	Coacervate-embedded gelatin-SH/PEGDA IPN hydrogels	Rabbit femoral trochlear osteochondral defect model	ADSCs and IGF-1 were transplanted with coacervate-embedded composite hydrogels	Dual delivery platform could induce chondrogenic differentiation of embedded ADSCs and promote osteochondral tissue regeneration	Cho et al. [123]
	Acellular cartilage extracellular matrix (ACECM) scaffolds	Pig osteochondral defect model	ADSCs combined with ACECM were transplanted into the defective area	ADSCs-ACECM composites successfully repaired the cartilage defect	Lu et al. [120]
Genetic modification	Chemically modified mRNA (modRNA)-mediated IGF-1 gene transfer	Mice osteoarthritis model	IGF-1-ADSCs were injected into the articular cavity	IGF-1-ADSCs decreased the loss of cartilage ECM, promoted cartilage repair, and exerted a chondroprotective effect	Wu et al. [68]
	ADSCs were transfected with miR-486-5p	Rat osteoarthritis model	Intra-articular injection of miR-486-5p-modified ADSC-Exos	miR-486-5p-modified ADSC-Exos attenuated chondrocyte apoptosis and alleviated osteoarthritis	Wang et al. [125]
Genetic modification + Bioscaffolds	Genetically engineered ADSCs overexpressing TGF-β1 (T-ADSCs); An injectable extracellular matrix (ECM)-mimicking hydrogel	Rat osteoarthritis model	T-ADSCs and hydrogels were injected into the articular cavity	T-ADSC-loaded hydrogels markedly reduced cartilage degeneration, joint inflammation, and subchondral bone loss	Yu et al. [67]
	ADSCs transfected with miR-99 b-3p-mimic; Hyaluronan-based hydrogel microparticles (HMPs)	Mice osteoarthritis model induced by surgical destabilization of the medial meniscus (DMM)	miR-99 b-3p-overexpressing ADSC-Exos were encapsulated in HMPs and injected into the articular cavity	miR-99 b-3p-modified ADSC-Exos combined with HMPs inhibited the degradation of the cartilage ECM	Yin et al. [126]

ADSCs have the potential for chondrogenic differentiation. The most characterized chondrogenic-inducing factors include TGF-β, fibroblast growth factor (FGF), IGF, and BMPs. These factors can be applied alone or in combination in the culture medium to promote the chondrogenesis of ADSCs [105, 106]. Moreover, SOX proteins are involved in chondrogenesis. The overexpression of SOX proteins enhances the chondrogenic differentiation of ADSCs and promotes cartilage healing [69]. In addition to chondrogenic differentiation, ADSCs contribute to cartilage regeneration via various mechanisms, including inhibiting chondrocyte pyroptosis [107], reducing chondrocyte reactive oxygen species production, suppressing inflammatory responses [108], and promoting ECM synthesis [21].

ADSCs exert cytoprotective, anti-inflammatory, and ECM synthetic effects on cartilage regeneration through paracrine mechanisms. The ADSC secretome can rectify abnormal osteoblast metabolism by suppressing the production of inflammatory mediators, such as IL-6 and prostaglandin E2 (PGE2) [109]. In an in vitro study, ADSC-EVs reduced the expression of pro-inflammatory cytokines and chemokines while inhibiting fibroblast-like synoviocyte-induced inflammation by interacting with the hyaluronan (HA) matrix and releasing miRNAs [110]. Another study confirmed that ADSCs-EVs inhibited chondrocyte matrix degradation and slowed osteoarthritis progression in a rat model by increasing and decreasing the expression of type II collagen and matrix metalloproteinases (MMPs), respectively [21]. Additionally, ADSC-derived microvesicles can suppress inflammation and modulate the metabolism of osteoarthritic synoviocytes [111]. In recent years, there has been a growing research interest in the use of ADSC-Exos for cartilage regeneration. Zhao et al. found that ADSC-Exos protected articular chondrocytes from H_2_O_2_-induced apoptosis and promoted chondrogenesis by upregulating miR-145 and miR-221 and downregulating pro-inflammatory cytokines, including IL-6 and TNF-α [53]. Another study demonstrated that ADSC-Exos could promote chondrocyte proliferation by miR-429 targeting FEZ2 and promoting autophagy [112]. Notably, hypoxic preconditioning of human ADSCs enhances their potential for chondrogenic differentiation [113]. Chang et al. demonstrated that hypoxia-cultured ADSC-secreted exosomes (hypoxia-ADSC-Exos) could enhance the synthesis of cartilaginous matrix and suppress the expression of inflammatory cytokines (TNF-α and IL-6) and degradation enzymes (MMP13 and ADAMT5) in vitro. Moreover, intra-articular treatment with hypoxia-ADSC-Exos exerted a chondroprotective effect by suppressing cartilage erosion, thereby slowing osteoarthritis progression [114]. Meng et al. harvested exosomes from tropoelastin-pretreated ADSCs and found that these exosomes effectively enhanced the matrix synthesis of chondrocytes in vitro and promoted cartilage repair in vivo [115]. These in vitro and in vivo experiments indicated that the ADSC secretome, particularly ADSC-Exos, is a promising option for treating cartilage lesions and osteoarthritis.

The combination of ADSCs with novel biomaterials or matrix proteins is being investigated as potential optimization strategies for cartilage regeneration. Biomaterials and matrix proteins can create a niche microenvironment that enhances the delivery, migration, proliferation, and differentiation of ADSCs [59]. As natural polymers, hyaluronic acid combined with allogeneic ADSCs efficiently promoted cartilage regeneration and prevented osteoarthritis progression in sheep [116, 117]. The amnion membrane (AM) from the human placenta was used to fabricate a minimally invasive injectable hydrogel as an ADSC carrier. Intra-articular injections of AM hydrogels with or without ADSCs alleviated inflammation and cartilage degeneration in a collagenase-induced osteoarthritis rat model, thus demonstrating the synergistic anti-inflammatory and chondroprotective effects of AM and ADSCs on cartilage tissues [18]. Najafi et al. fabricated a composite scaffold containing ECM powder and several layers of hydrogel and nanofibers to mimic the structure and characteristics of cartilage and subsequently encapsulated human ADSCs within this scaffold [118]. The composite scaffold improved cell proliferation and enhanced the chondrogenic-like differentiation of ADSCs. In addition, studies have revealed the beneficial effects of an injectable hydrogel consisting of glycol chitosan/dibenzaldehyde-terminated polyethylene glycol [119] or acellular cartilage ECM scaffolds [120] combined with ADSCs on cartilage tissue repair and regeneration.

Notably, severe cartilage lesions can also compromise the subchondral bone. Subsequently, osteochondral regeneration strategies must be employed to restore both the zonal articular cartilage and the subchondral bone using three elements: scaffolds, cells, and bioactive factors. These components are being employed for structural reconstruction and tissue activity remodeling [121]. Zhang et al. fabricated a composite scaffold consisting of hyaluronic acid, chitosan, and PLGA with two different sites: one designed to support hyaline chondrogenesis and the other designed to support osteogenesis by binding to BMP-2. These osteochondral scaffolds were seeded with ADSCs, resulting in the regeneration of rabbit osteochondral defects [122]. Cho et al. fabricated a coacervate-embedded gelatin-SH/PEGDA IPN hydrogel for the co-delivery of ADSCs and chondrogenic factor IGF-1 and demonstrated that this dual delivery system could induce chondrogenic differentiation of the embedded ADSCs and effectively augment osteochondral tissue regeneration [123]. Furthermore, 3D printing is advantageous for producing scaffolds with customized shapes and structures/composition gradients. Seeding multiphasic 3D-bioprinted scaffolds with human ADSCs could facilitate the site-specific chondrogenic and osteogenic differentiation of ADSCs in vitro [124]. However, this advanced bioengineering strategy for cartilage regeneration requires further in vivo validation.

Genetic modifications have been used to optimize ADSC-based therapies for cartilage regeneration. Wu et al. transfected ADSCs with IGF-1-modified mRNA (termed IGF-1-ADSCs) to enhance chondrogenesis and found that IGF-1-ADSCs increased anabolic marker expression in chondrocytes in vitro, decreased cartilage ECM loss, and promoted cartilage repair in vivo [68]. Additionally, Yu et al. applied genetically engineered ADSCs overexpressing TGF-β1 (termed T-ADSCs) to exert anti-inflammatory and pro-anabolic effects on chondrocytes and confirmed that T-ADSC-loaded hydrogels markedly reduced cartilage degeneration, joint inflammation, and subchondral bone loss in vivo [67]. In another study, miR-486-5p-modified ADSC-Exos modulated endoplasmic reticulum stress, attenuated chondrocyte apoptosis in vitro, and alleviated osteoarthritis in vivo [125]. The overexpression of mir-99 b-3p in exosomes extracted from subcutaneous adipose tissue-derived stem cells (Sc-ADSCs-Exos) could enhance the therapeutic effect of inhibiting cartilage ECM degradation and promoting cartilage repair. Moreover, the encapsulation of these genetically engineered Sc-ADSCs-Exos in an HA-based hydrogel microparticle scaffold provided for sustained local drug release and simultaneously strengthened the chondroprotective effects [126].

The efficacy and safety of intra-articular ADSC injection for patients with knee osteoarthritis have been confirmed clinically, with this treatment leading to functional improvement, pain relief, cartilage restoration, and retardation of disease progression, as measured via magnetic resonance imaging (MRI), with no serious adverse events [127–132]. In addition, the intra-articular injection of autologous adipose-derived SVFs (ADSVFs) in patients with knee osteoarthritis has yielded good results [133–136]. Although the evidence is limited, the conclusions of these studies suggest that ADSCs or ADSVFs, which are rich in ADSCs, exert promising effects and are considered safe as an alternative treatment option for osteoarthritis. However, further large-scale randomized controlled trials and long-term follow-up studies are warranted to validate their clinical use.

Unlike articular cartilage, which is a smooth elastic tissue covered with hyaline cartilage on the joint surface, the intervertebral disk (IVD) is a fibrocartilaginous tissue that consists of inner soft nucleus pulposus (NP) cells, the surrounding annulus fibrosus, and cartilaginous endplates. IDD is the most common musculoskeletal disorder in the elderly and involves several endogenous molecular processes, including ECM degeneration, senescence, apoptosis, oxidative stress, inflammation, and reduced autophagy, which is similar to the effects of osteoarthritis on the facet joint of the spine [137]. Current evidence indicates that ADSC-based strategies have promising effects on IDD. Yu et al. used a genipin-crosslinked decellularized NP hydrogel (GDH) as a novel system to load ADSCs and observed that GDH could promote the differentiation of ADSCs into NP cells while enhancing the intervertebral height, MRI index, and histological grading scores in vivo [138]. In addition, Xing et al. demonstrated that the injection of ECM acellular biological scaffolds loaded with ADSC-Exos could regulate IVD microenvironment homeostasis and ameliorate IDD [139]. However, additional studies are warranted to explore and verify the effects of ADSC-based strategies on human IDD.

### Tendon regeneration

The tendon is a well-organized, dense connective tissue that connects the muscle to the bone and transmits force, thereby enabling motion and posture maintenance. Tendon injuries are the most common injuries of the musculoskeletal system, and they are frequently caused by overloading and characterized by tendon swelling, local pain, and disability. No current therapeutic approach for tendon injuries provides a successful, long-term solution. Furthermore, owing to the inferior regenerative capacity of tendons, repaired tendons cannot restore their pre-injury strength and function, leading to degenerative changes and a high risk of re-injury. Tendon tissue engineering involving ADSCs is expected to promote tendon regeneration and reconstruction (Table 3).

**Table 3:** Recent preclinical studies on ADSC-based optimization strategies for tendon regeneration

ADSC-based optimization strategies		Models	Methods	Results	References
ADSCs	ADSCs preconditioned with GDF-6 and PDGF-BB; collagen/alginate gel	Rat model of Achilles tendon excision defect	Tenogenically differentiated ADSCs with the hydrogel were injected into the defective area	Tenogenically differentiated ADSCs enhanced the collagen fiber dispersion range closest to the normal tendon	Norelli et al. [147]
	ADSCs induced by GDF-5 and PDGF; collagen/alginate gel	Rat model of Achilles tendon defect	ADSCs pre-treated with hydrogel were injected into the tendon defect area	GDF5/PDGF-induced ADSCs promoted tendon repair by improving cellular proliferation, tenogenesis, and vascular infiltration	Fitzgerald et al. [142]
Engineered ADSCs	ADSC sheets	Rat model of chronic rotator cuff tear	ADSC sheets were transplanted into the rotator cuff tear area	ADSC sheets significantly enhanced the biomechanical properties of the repaired rotator cuff	Shin et al. [148]
Engineered ADSCs + Bioscaffolds	ADSC sheets (P(LLA-CL)/Silk fibroin nanoyarn scaffolds	Rabbit model of patellar tendon defect	GDF-5-induced ADSC sheets were seeded on nanoyarn scaffolds and implanted into the patellar tendon defect area	GDF-5-induced ADSC sheets stimulated higher expression of tenogenesis-related markers and promoted functional tendon regeneration	Chen et al. [28]
Bioscaffolds	Fibrin or gelatin methacrylate [GelMA]	Rat model of massive rotator cuff tears	ADSCs seeded in fibrin or GelMA hydrogel were implanted into the repair site	ADSCs combined with fibrin or GelMA hydrogel could decrease bone loss and augment the efficacy of surgical repair	Rothrauff et al. [150]
	Novel injectable porous gelatin microcryogels (GMs)	Rat model of acute Achilles tendon rupture	GMs loaded with ADSCs were injected into the gap	ADSCs with GMs could effectively improve the macroscopic appearance, histological morphology, and biomechanical properties of the repair tissue	Yang et al. [151]
ADSC-Exos		Rat model of a massive rotator cuff tear	ADSC-Exos were injected into the damaged site	ADSC-Exos treatment could prevent atrophy, fatty infiltration, and inflammation and promote myofiber regeneration and the biomechanical properties of the injured rotator cuff	Wang et al. [155]
		Rabbit model of chronic rotator cuff tear	ADSC-Exos were injected into the repair site	ADSC-Exos could prevent fatty infiltration, improve biomechanical properties, and promote tendon–bone healing after surgical repair	Wang et al. [54]
ADSC-Exos + Bioscaffolds	Hydrogel	Rat model of rotator cuff injury	ADSC-Exos-hydrogel complex was injected in the shoulder after surgical repair	ADSC-Exos-hydrogel promoted rotator cuff repair by mediating the differentiation of the tendon-derived stem cells	Fu et al. [156]
	In situ-forming fibrin gel	Rabbit model of partial-thickness rotator cuff tears	Local administration of in situ-forming fibrin gel containing ADSC-Exos (ADSC-Exos/fibrin)	ADSC-Exos/fibrin significantly prevented tear progression, enhanced the biomechanical properties of the injured tendon, and promoted high-quality tendon healing	Wang et al. [157]

ADSCs can differentiate into tenocytes to repair the injured tendon. Several growth factors have been utilized to differentiate stem cells into tendon-like cells in vitro, including IGF-1, TGF-β, FGF, PDGF, growth differentiation factor (GDF)-5, and connective tissue growth factor [140–142]. Hypoxic preconditioning can enhance the expression of tendon-related markers and has been proposed to stimulate the tenogenic differentiation of ADSCs [143]. In addition, ECM-based approaches can be utilized for tendon regeneration. Rao et al. prepared a soluble, DNA-free, bovine tendon-derived ECM using a urea-based method, and it showed a strong pro-tenogenic capacity on human ADSCs [20].

Behfar et al. confirmed that the intratendinous injection of ADSVFs enriched with ADSCs improved the mechanical properties of the injured tendon, thereby increasing yield loads and energy absorption [144]. Local injection of ADSCs improved muscle function and tendon healing and decreased fatty infiltration in a chronic rotator cuff tear rabbit model [145], and it also increased the expression of collagen I and collagen III following Achilles tendon rupture in rabbits [146]. Norelli et al. explored the efficacy of ADSCs with or without tenogenic differentiation induced by GDFs and PDGF-BB in vivo and found that ADSCs improved the biomechanical properties of repaired tendons, and tenogenically differentiated ADSCs could enhance the mean histological score and collagen fiber dispersion range closest to the normal tendon [147]. Another study demonstrated that pre-conditioned ADSCs with GDF-5 and PDGF increased the expression of the protenogenesis gene *SOX9* and promoted cell–cell connections and cellular proliferation, thereby accelerating the remodeling process of the injured tendon [142].

Cell sheet technology is an innovative tool for tissue regeneration that preserves intact cell–cell connections and the ECM. Shin et al. confirmed the efficacy of ADSC sheets as a viable cell delivery tool for tendon reconstruction [148]. In their study, ADSC sheets were fabricated using a temperature-responsive dish and transplanted into the defective area during repair procedures in a rat rotator cuff tear model. The ADSC sheets significantly enhanced the biomechanical properties of the repaired rotator cuff. In another study, Chen et al. observed that GDF-5-induced ADSC sheets combined with nanoyarn scaffolds could stimulate higher expression of tenogenesis-related markers and promote functional tendon regeneration [28]. These findings suggest that engineered ADSC sheets can be used in tendon repair and regeneration.

Novel scaffold materials could facilitate the engraftment and differentiation of ADSCs while promoting tendon healing. Chiou et al. confirmed that supplementing a biocompatible tendon hydrogel with platelet-rich plasma and ADSCs could promote early mechanical strength and functional restoration, thereby accelerating injured tendon repair [149]. Rothrauff et al. reported that fibrin- or gelatin methacrylate-seeded ADSCs could decrease bone loss and promote surgical repair efficacy in rats with massive rotator cuff tears [150]. Injectable porous gelatin microcryogels (GMs) promoted ADSC proliferation and facilitated ECM secretion, and ADSC-GM matrices exhibited regeneration and repair potential for Achilles tendon ruptures in rats [151]. Guo et al. used the small intestinal submucosa (SIS) as a scaffold to seed ADSCs and repair Achilles tendon defects in a rat model. Engineered tendon grafts created by seeding ADSCs on SIS could significantly improve ECM production and collagen fiber compactness, thus allowing for a higher peak tensile load of the restored Achilles tendon [143]. Recently, a functionally graded 3D scaffold was fabricated by incorporating platelet lysate within an electrospun fiber core. These 3D functional scaffolds featuring gradients in composition and topography promoted the proliferation and differentiation of ADSCs and induced the regeneration of different elements, which exhibited the hierarchical structure of the tendon-bone interface [152]. However, in vivo studies using clinical tendon injury models are warranted to evaluate the regenerative performance of these 3D functional gradient scaffolds.

Exosomes, as a kind of cell-free therapeutic strategy, may provide new perspectives for tendon-bone healing [153]. ADSC-Exos could decrease the expression of pro-inflammatory cytokines, maintain metabolic homeostasis, and improve the histological properties of injured tendons in vitro [154]. Wang et al. [155] demonstrated that ADSC-Exos effectively decreased atrophy and degeneration while improving the myofiber regeneration and biomechanical properties of torn rotator cuff muscles. The ADSC-Exos could also prevent fatty infiltration, promote tendon-bone healing, and improve biomechanical properties in a rabbit model of chronic rotator cuff tears [54]. Hydrogels can provide mechanical support and promote the sustained release of ADSC-Exos. Fu et al. injected an ADSC-Exos-hydrogel complex into the shoulders of rats in a rotator cuff injury model and observed enhanced rotator cuff repair and tendon-bone healing attributed to ADSC-Exos promoting the differentiation of tendon-derived stem cells [156]. Wang et al. [157] demonstrated that the local administration of a fibrin gel containing ADSC-Exos could significantly prevent tear progression, enhance the biomechanical properties of the injured tendon, and promote high-quality tendon healing in a rabbit model of partial-thickness rotator cuff tears.

To date, only a few clinical trials have been conducted using ADSCs to treat tendon injuries. One study confirmed the beneficial effect of injecting ADSCs loaded with fibrin glue during arthroscopic rotator cuff repair on structural integrity, as assessed by MRI [158]. Although no clinical differences were observed during the 28-month follow-up period, the results indicated that ADSCs provided an adequate biological environment around the tendon repair site. Furthermore, Jo et al. demonstrated that the intra-tendinous injection of autologous ADSCs in patients with rotator cuff injuries could significantly reduce shoulder pain, increase muscle strength, improve shoulder function, and reduce bursal-sided defect volumes without treatment-related adverse events at a minimum follow-up of 2 years [159, 160]. In addition, Hurd et al. observed that fresh, uncultured, unmodified, autologous adipose-derived regenerative cells isolated from lipoaspirate were more effective than corticosteroid injection and could improve shoulder function without adverse effects [161]. Recently, Randelli et al. applied autologous microfragmented lipoaspirate tissue containing ADSCs to treat patients with degenerative posterosuperior rotator cuff tears and observed that the intraoperative injection of autologous microfragmented adipose tissue could effectively improve the function of rotator cuff repair [162]. Collectively, the abovementioned clinical findings indicate the beneficial effects of ADSCs on rotator cuff tears. However, larger samples and long-term follow-up studies are required to validate these findings.

## Conclusions and perspectives

ADSCs and ADSC-based optimization strategies represent promising therapeutic tools in the fields of tissue engineering and regenerative medicine. Preclinical studies have revealed the therapeutic function of ADSC-based strategies in bone, cartilage, and tendon regeneration. Furthermore, other properties of ADSCs, such as pro-angiogenic, anti-inflammatory, anti-apoptotic, and pro-ECM synthesis, have been demonstrated both in vitro and in vivo. Finally, the findings of preliminary clinical trials indicate the beneficial effects of ADSCs in promoting musculoskeletal tissue repair and reconstruction (Table 4).

**Table 4:** Recent clinical trials on ADSC-based therapies for musculoskeletal regeneration

Application	Participants	Methods	Follow-up time	Results	References
Bone regeneration	13 patients with craniomaxillofacial hard-tissue defects	Autologous ADSCs were seeded onto either bioactive glass or β-tricalcium phosphate scaffolds and transplanted into the bone defect area	12–52 months	In 10 of 13 cases, the construct was successfully integrated into the surrounding skeleton	Sándor et al. [101]
	5 cranial defect patients	Patients received cranioplasties using autologous ADSCs, beta-tricalcium phosphate granules, and supporting meshes	6 years	Long-term clinical results were not satisfactory, partially owing to graft resorption, tumor recurrence, or late infection	Thesleff et al. [103]
Cartilage regeneration	12 patients with knee osteoarthritis	Autologous ADSCs were intra-articularly administered	6 months	ADSC injection provided significant functional improvement and pain relief	Lee et al. [127]
	53 patients with symptomatic knee osteoarthritis	Autologous adipose-derived mesenchymal progenitor cells (haMPCs) expanded in vitro were intra-articularly injected	12 months	Significant improvements in joint function, pain, quality of life, and cartilage regeneration with good tolerance	Lu et al. [128]
	30 patients with symptomatic knee osteoarthritis	Intra-articular ADSC therapy	12 months	Autologous ADSC therapy is safe and effective and can prevent disease progression	Freitag et al. [129]
	18 patients with knee osteoarthritis	Autologous ADSCs were intra-articularly injected	96 weeks	Autologous ADSCs improved the pain, function, and cartilage volume of the knee joint	Song et al. [131]
Tendon regeneration	70 patients with full-thickness rotator cuff tear	35 patients underwent arthroscopic rotator cuff repair with autologous ADSC injections, whereas 35 patients underwent only repair surgery	At least 12 months	ADSC injection during rotator cuff repair significantly decreased the retear rate; furthermore, the function of the repaired tissue was similarly ameliorated in both groups	Kim et al. [158]
	18 patients with partial-thickness rotator cuff tear	Intratendinous injection of autologous ADSCs	2 years	Autologous ADSC injection improved shoulder function, relieved pain, and exhibited continued safety and efficacy	Jo et al. [160]
	11 patients with partial-thickness rotator cuff tears	Autologous adipose-derived regenerative cells were injected into the shoulder with a single injection	12 months	Autologous adipose-derived regenerative cell injection improved shoulder function without adverse effects	Hurd et al. [161]
	44 patients with degenerative posterosuperior rotator cuff tear	22 patients underwent arthroscopic rotator cuff repair augmentation with autologous microfragmented lipoaspirate tissue, whereas 22 patients underwent only repair surgery	24 months	Injection of autologous microfragmented adipose tissue effectively promoted functional rotator cuff repair	Randelli et al. [162]

However, concerns such as limited cell survival, senescence-induced genetic instability, functional inactivation, the possibility of unfavorable ADSC differentiation, and the relatively low purity and yield of ADSC-Exos must be addressed before applying ADSCs and their cell-free derivatives. The intrinsic characteristics of donors (e.g., age, sex, and obesity), differences in adipose tissue sources, and differences in the isolation procedures can affect the properties of ADSCs or ADSC-Exos. These limitations indicate the need for quality control of ADSCs and ADSC-Exos. In addition, further exploration of appropriate scaffolds, potent bioactive factors, and genetically modified approaches is required to provide more optimized conditions for the proliferation and differentiation of ADSCs and verify their efficacy and safety in humans. Considering the differences between preclinical studies and clinical trials, the oncogenicity of ADSC differentiation should be further investigated. ADSC-Exos can avoid many of the shortcomings associated with administering ADSCs, including potential tumorigenicity and storage problems, and thus may represent an effective regenerative agent for musculoskeletal regeneration. However, limited cellular and animal experiments have been performed. Therefore, large-scale clinical trials are urgently warranted.

Despite the current challenges, the significant progress achieved in this field suggests that ADSC-based therapies will play increasingly crucial roles in tissue regeneration. Technological improvements and additional investigations may help gradually tackle these problems. Researchers can harvest suitable ADSC subpopulations using standardized isolation procedures and then enhance the therapeutic potential of these cells by preconditioning them with bioactive factors or combining them with biomaterials before transplantation. Nevertheless, larger prospective, blinded, randomized clinical trials are warranted to further establish the long-term effectiveness and safety of ADSC-based therapies in humans. Such work may result in a paradigm shift in the treatment of musculoskeletal disorders.

## Data Availability

Not applicable.

## Abbreviations

ADSCs: Adipose-derived stem cells
ADSC-Exos: ADSC-derived exosomes
ADSC-CM: ADSC-conditioned medium
AM: Amnion membrane
BM: Bone marrow
BMP: Bone morphogenetic protein
CD: Cluster of differentiation
3D: Three-dimensional
ECM: Extracellular matrix
EVs: Extracellular vesicles
GA: Gallic acid
GDF: Growth differentiation factor
GDH: Genipin-crosslinked decellularized nucleus pulposus hydrogel
GMs: Gelatin microcryogels
HA: Hyaluronan
HLA: Human leukocyte antigen
HSP: Heat shock proteins
IDD: Intervertebral disk degeneration
IFATS: International Federation for Adipose Therapeutics and Science
IGF-1: Insulin-like growth factor 1
IL: Interleukin
ISCT: International Society for Cellular Therapy
IVD: Intervertebral disk
MBG: Mesoporous bioactive glass
miRNAs: MicroRNAs
MMP: Matrix metalloproteinases
MOF: Metal–organic framework
MRI: Magnetic resonance imaging
MSCs: Mesenchymal stem cells
ncRNAs: Noncoding RNAs
NP: Nucleus pulposus
PGE2: Prostaglandin E2
PLA: Polylactic acid
PLGA: Polylactic-co-glycolic acid
PLGA/pDA: Polydopamine-coating poly lactic-co-glycolic acid
SIS: Small intestinal submucosa
SOX: SRY (sex-determining region Y) box containing gene
SVF: Stromal vascular fraction
TCP: Tricalcium phosphate
β-TCP: β-Tricalcium phosphate
TGF-β: Transforming growth factor-β
TNF: Tumor necrosis factor
TSG: Tumor suppressor gene
VEGF: Vascular endothelial growth factor
